# Assessment of Oxidative Stress and Neuronal Damage Indicators After Parathyroidectomy in Patients With Primary Hyperparathyroidism

**DOI:** 10.7759/cureus.109915

**Published:** 2026-05-30

**Authors:** Uğur Özberk, İncilay Lay, Ahmet Yılmaz, Süleyman N Şendur, Ömer A Gürlek

**Affiliations:** 1 Internal Medicine, Hacettepe University School of Medicine, Ankara, TUR; 2 Biochemistry, Hacettepe University School of Medicine, Ankara, TUR; 3 Central Laboratory, Hacettepe University Hospitals, Ankara, TUR; 4 Endocrinology and Metabolism, Hacettepe University School of Medicine, Ankara, TUR

**Keywords:** neurofilament proteins, neuronal damage, oxidative stress, parathyroidectomy, primary hyperparathyroidism

## Abstract

Background: Primary hyperparathyroidism (PHPT) is associated with neurological damage, which tends to improve after parathyroidectomy (PTX). Oxidative stress might play a role in these pathological changes. This study aimed to investigate oxidative stress and neuronal damage markers following PTX in patients with PHPT.

Methods: The study involved 36 patients with PHPT who underwent surgical treatment. Markers for oxidative stress, including serum total antioxidant status (TAS), total oxidative status (TOS), oxidative stress index (OSI), 7-ketocholesterol (7-KC) and cholestan-3β,5α,6β-triol (C-triol) as oxysterols and 8-iso-prostaglandin F2α (8-iso-PGF2α), were measured, along with neurofilament light chain (NfL) as a marker for neuronal damage. These markers were assessed before and six months after PTX.

Results: Following PTX, a significant reduction in OSI was observed (*p* = 0.020), while TAS, TOS, oxysterol, 8-iso-PGF2α, and NfL levels remained unchanged. There was no correlation between baseline serum calcium and parathyroid hormone levels with baseline oxidative stress markers or NfL levels. However, baseline TAS levels showed a correlation with baseline NfL levels (*r* = −0.370), which was not present after surgery.

Conclusions: These findings suggest oxidative stress is elevated in patients with PHPT and may contribute to neurological complications. Post surgery, oxidative stress levels decrease, as reflected by a significant reduction in OSI; whether this translates into better central nervous system outcomes warrants confirmation in future studies.

## Introduction

Primary hyperparathyroidism (PHPT) is a frequently encountered endocrine disease characterized by elevated serum calcium and parathyroid hormone (PTH) concentrations. Traditionally, this condition has been associated with complications including kidney stones, osteoporosis, and symptoms related to hypercalcemia [1]. However, recent diagnoses were often made through routine screenings revealing high blood calcium levels. While many patients present with asymptomatic hypercalcemia, PHPT can affect a wide range of cells, including neurons [2]. 

Reactive oxygen species (ROS), such as hydrogen peroxide, hydroxyl radicals, and superoxide anion radicals, are unstable oxygen-containing molecules produced during normal metabolism that can impair vital cellular components. To counteract this damage, the body relies on antioxidant defense systems. An imbalance favoring ROS accumulation over the body's antioxidant defenses results in oxidative stress. Various diseases, particularly neurodegenerative disorders, have been linked to the pathogenic effects of oxidative stress [3-6].

Markers such as total oxidative status (TOS) and total antioxidant status (TAS) are utilized to evaluate oxidative stress. TOS indicates the overall oxidative state, while TAS measures overall antioxidant levels [7,8]. The oxidative stress index (OSI) provides a comprehensive assessment by calculating the ratio of TOS to TAS. Cholestan-3β,5α,6β-triol (C-triol) and 7-ketocholesterol (7-KC) are oxysterols formed through oxidative processing of cholesterol that serve as markers of oxidative stress [9,10]. Oxidative stress may also be evaluated through the measurement of F2-isoprostanes, products of arachidonic acid oxidation, with 8-iso-prostaglandin F2α (8-iso-PGF2α) being the most commonly measured [11]. 

Neurofilaments are cytoskeletal proteins specific to neurons that support their structural integrity. Neurofilament light chain (NfL), a component of these proteins, is predominantly found in the neuronal cytoplasm [12]. Typically, low concentrations of NfL are continuously released by neurons, but its release increases in response to axonal damage [13]. 

Given that central nervous system involvement can occur in PHPT, it is plausible that oxidative stress markers and NfL levels might be altered in these patients. There are very few studies related to TAS, TOS, and OSI in PHPT [14,15], and no study has been conducted in PHPT using oxysterols, 8-iso-PGF2α, or NfL. We therefore hypothesized that successful parathyroidectomy (PTX) would lead to a reduction in oxidative stress markers and NfL levels, and aimed to determine whether PTX attenuates systemic oxidative stress and neuronal injury in patients with PHPT, with changes in oxidative stress markers (TAS, TOS, OSI, oxysterol profiles, and 8-iso-PGF2α) and neuronal damage (NfL) as primary outcomes.

## Materials and methods

The study was designed as a single-arm prospective pre-post investigation; no external comparator group was included. It was conducted at a tertiary referral hospital in Ankara, Turkey. The Hacettepe University Clinical Research Ethics Committee approved the study (approval number: GO 21/784; approval date: 29/06/2021), and the study was conducted in line with the ethical standards of the Declaration of Helsinki. All participants provided written informed consent prior to their inclusion in the study.

Study population

Patients with PHPT who visited our clinic from August 2021 to October 2023 were recruited for the study. The study population consisted of 36 consecutive patients eligible for PTX according to the latest guidelines. Patients with neurological or psychiatric disorders, persistent disease, chronic kidney disease, heart failure, or chronic liver disease were excluded. Persistent disease was defined as failure to normalize calcium levels within six months post-surgery.

Data collection and interventions

Demographic data (age and gender) of the patients were recorded. Body weight and height were recorded before the surgical procedure, and body mass index (BMI) was computed by dividing weight in kilograms by the height in metres squared (kg/m²). Blood pressure (BP) was measured after at least 15 minutes of seated rest. Nephrolithiasis and osteoporosis were assessed using ultrasonography and bone mineral densitometry, respectively. Diabetes mellitus, hypertension, and hyperlipidemia were diagnosed following international guidelines [16-18].

All patients underwent minimally invasive parathyroidectomy. No vitamin D supplementation was initiated postoperatively. Concomitant medications were kept unchanged throughout the study period to minimize the potential confounding effects of medication changes on oxidative stress and neuronal damage markers.

Fasting blood samples were obtained from patients both before and six months following PTX using Vacutainer tubes containing a clot activator. Samples were spun at 3000 rpm for 10 minutes and kept at −80 °C until further processing. Samples were tested for calcium, phosphorus, PTH, 25-hydroxyvitamin D (25(OH)D), glucose, hemoglobin A1c (HbA1c), total cholesterol, high-density lipoprotein (HDL) cholesterol, low-density lipoprotein (LDL) cholesterol, very-low-density lipoprotein (VLDL) cholesterol, triglycerides, and creatinine. The Chronic Kidney Disease Epidemiology Collaboration (CKD-EPI) equation was employed to calculate estimated glomerular filtration rates (eGFR) [19].

TAS was quantified using a colorimetric assay (E-BC-K801-M; Elabscience Bionovation Inc., Houston, Texas, United States). This assay evaluates the antioxidant capacity of the sample by determining its ability to prevent the oxidation of 2,2'-azino-bis(3-ethylbenzothiazoline-6-sulfonic acid) (ABTS) into its radical cation form, ABTS•+. In the presence of antioxidants, ABTS•+ is reduced to colorless ABTS, while ABTS is oxidized to a green ABTS•+ radical by an oxidant. The TAS was determined by measuring the absorbance of ABTS•+ at 660 nm. Trolox was used as a standard to quantify antioxidant levels, and the findings were reported in mmol Trolox equivalent/L. The detection range of the assay was 0.23-2 mmol Trolox Equiv./L, with intra-assay and inter-assay coefficients of variation (CV) of 4.6% and 7%, respectively.

TOS was also measured using a colorimetric assay (E-BC-K802-M; Elabscience Bionovation Inc.). This assay operates on the principle that under acidic conditions, oxidants in the sample convert Fe2+ to Fe3+, which generates a blue-purple complex with xylenol orange. The maximum absorbance of this complex occurs around 590 nm. The color intensity correlates with the concentration of oxidants in the sample, allowing for calculation of the total oxidation state. Hydrogen peroxide (H2O2) was used as a standard for quantification, and results were expressed in μmol H2O2 equivalent/L. The detection range was 2.5-100 μmol H2O2 Equiv./L, with intra-assay and inter-assay CVs of 2.3% and 3.5%, respectively.

The OSI was calculated by dividing TOS by TAS, providing a measure of oxidative stress. The formula for OSI is: $\text{OSI (arbitrary units)} = \frac{\text{TOS } (\mu\text{mol H}_2\mathrm{O}_2 \text{ Eq/L})}{\text{TAS } (\text{mmol Trolox Eq/L})}$

Plasma concentrations of 7-KC and C-triol were determined using LC-MS/MS (QTRAP 4500; SCIEX Corporation, Toronto, Canada) according to the protocol described by Jiang et al. [20]. Briefly, plasma samples (50 µL) were subjected to protein precipitation with methanol containing deuterium-labeled internal standards ([²H₇]7-ketocholesterol and [²H₇]cholestane-3β,5α,6β-triol), followed by derivatization with N,N-dimethylglycine to enhance ionization efficiency. Chromatographic separation was performed on a Betasil C18 column (Thermo Fisher Scientific Inc., Waltham, Massachusetts, United States), and detection was carried out in multiple reaction monitoring (MRM) mode using an atmospheric pressure chemical ionization (APCI) source in positive ion mode. The lower limit of quantification (LLOQ) was 2 ng/mL for both analytes. Intra-run and inter-run precision (CV) were less than 15% across all quality control levels, and extraction recoveries were 89% for C-triol and 90% for 7-KC. 

Serum 8-iso-prostaglandin F2α (8-iso-PGF2α) levels were measured using a human enzyme-linked immunosorbent assay (ELISA) kit (CEA701Ge; Cloud-Clone Corp., Houston, Texas, United States). The detection range of the assay was 24.69-2,000 pg/mL, with a sensitivity of 9.11 pg/mL, and intra-assay and inter-assay CVs of less than 10% and 12%, respectively. Serum neurofilament light chain (NfL) levels were measured using a high-sensitivity human ELISA kit (HEE038Hu; Cloud-Clone Corp.). The detection range was 15.6-1,000 pg/mL, with a sensitivity of 6.3 pg/mL, and intra-assay and inter-assay CVs of less than 10% and 12%, respectively. Optical density was read at 450 nm for both ELISA assays.

Statistical analyses

The Shapiro-Wilk test was conducted to determine whether the variables followed a normal distribution. Categorical data were displayed as frequencies and percentages (%). Continuous data exhibiting a normal distribution were reported with their mean and standard deviation (SD), while non-normally distributed variables were summarized using median (range). Pre- and postoperative comparisons were performed using the paired-samples t-test for normally distributed variables (calcium, phosphorus, creatinine, eGFR, total cholesterol, triglycerides, HDL, LDL, VLDL, TAS, and OSI) and the Wilcoxon signed-rank test for non-normally distributed variables (PTH, 25(OH)D, fasting plasma glucose, HbA1c, TOS, 7-KC, C-triol, 8-iso-PGF2α, and NfL). Results were reported as t-statistic with degrees of freedom (df = n − 1) for paired t-test and W statistic for Wilcoxon signed-rank test, alongside p values. To evaluate relationships between variables, Pearson or Spearman correlation analyses were performed according to the underlying data distribution. Since at least one variable in each correlation pair did not meet the assumption of normality, Spearman correlation analysis was used for all correlations. Results are reported as Spearman correlation coefficient (R) with degrees of freedom (df = n − 2) and p values. A threshold of p < 0.05 was used to establish statistical significance. Missing data were present only for osteoporosis (n=5) and nephrolithiasis (n=2), and were handled using complete-case analysis; these patients were excluded only from the analysis of the respective variable and retained for all other analyses. Statistical analyses were performed using IBM SPSS Statistics for Windows, version 25 (IBM Corp., Armonk, New York, United States).

## Results

Of the 36 patients, 27 (75%) were female and nine (25%) were male, with a mean age of 55.75 years (Table 1). Ten patients (27.8%) were current smokers, three patients (8.3%) were former smokers, and the remaining 23 (63.9%) were non-smokers. Most patients presented with asymptomatic hypercalcemia and had mild PHPT. Evaluation for PHPT revealed that 11 patients (35.5%) had osteoporosis and nine patients (26.5%) had nephrolithiasis. Data on osteoporosis were missing for five patients and on nephrolithiasis for two patients because these individuals had undergone surgery before their PHPT evaluations were completed. Additionally, 25 patients (69.4%) had hypertension, eight patients (22.2%) had diabetes, and 20 patients (55.6%) had hyperlipidemia. 

**Table 1:** Demographic and clinical features of the patients (N=36) ^†^ Data available for 31 patients; five patients underwent surgery before bone mineral densitometry was completed. ^‡^ Data available for 34 patients; two patients underwent surgery before renal ultrasonography was completed. BMI: body mass index

Parameters	Values
Age (years), mean ± SD	55.75 ± 13.80
Sex, n (%)	
Female	27 (75%)
Male	9 (25%)
Height (cm), mean ± SD	163.44 ± 9.83
Weight (kg), mean ± SD	76.75 ± 12.67
BMI (kg/m²), mean ± SD	28.94 ± 5.51
Smoking status, n (%)	
Active smoker	10 (27.8%)
Ex-smoker	3 (8.3%)
Non-smoker	23 (63.9%)
Clinical presentation, n (%)	
Asymptomatic hypercalcemia	33 (91.7%)
Symptomatic nephrolithiasis	3 (8.3%)
Osteoporosis	11/31 (35.5%)†
Nephrolithiasis	9/34 (26.5%)‡
Hypertension	25 (69.4%)
Diabetes mellitus	8 (22.2%)
Hyperlipidemia	20 (55.6%)

Following PTX, serum calcium and PTH levels returned to normal (t = 11.018, p < 0.001; W = 7.0, p < 0.001, respectively), while serum phosphorus and 25(OH)D levels increased significantly (t = −6.701, p < 0.001; W = 146.5, p = 0.003, respectively). Despite these changes, serum TAS (t = −1.224, p = 0.231), TOS (W = 112.5, p = 0.060), 7-KC (W = 216.0, p = 0.085), C-triol (W = 49.0, p = 0.388), 8-iso-PGF2α (W = 301.0, p = 0.610), and NfL (W = 261.0, p = 0.250) levels remained stable, whereas OSI decreased significantly (t = 2.388, p = 0.020). Other laboratory parameters did not show significant changes after surgery (Table 2).

**Table 2:** Biochemical data of the patients before and six months after parathyroidectomy t = t statistic; df = 35 (n = 36, df = n − 1); W = Wilcoxon signed-rank test statistic; degrees of freedom are not applicable for nonparametric tests. PTH: parathyroid hormone, 25(OH)D: 25-hydroxyvitamin D, eGFR: estimated glomerular filtration rate, HbA1c: hemoglobin A1c, HDL: high-density lipoprotein, LDL: low-density lipoprotein, VLDL: very low-density lipoprotein, TAS: total antioxidant status, TOS: total oxidative status, OSI: oxidative stress index, AU: arbitrary units, 7-KC: 7-ketocholesterol, C-triol: cholestan-3β,5α,6β-triol, 8-iso-PGF2α: 8-iso-prostaglandin F2α, NfL: neurofilament light chain.

Parameters	Preoperative values	Postoperative values	Test statistic		P value
Calcium (mg/dL), mean ± SD	10.86 ± 0.77	9.41 ± 0.37	t = 11.018	< 0.001	
PTH (pg/mL), median (range)	153.5 (45.1-478)	63 (18-139)	W = 7.0	< 0.001	
Phosphorus (mg/dL), mean ± SD	2.73 ± 0.54	3.34 ± 0.55	t = −6.701	< 0.001	
25(OH)D (µg/L), median (range)	17 (4-38)	21.5 (7.9-45.9)	W = 146.5	0.003	
Creatinine (mg/dL), mean ± SD	0.68 ± 0.14	0.70 ± 0.10	t = −1.243	0.220	
eGFR (mL/min/1.73 m²), mean ± SD	103.20 ± 14.05	102.21 ± 13.28	t = 0.842	0.409	
Fasting plasma glucose (mg/dL), median (range)	97 (78-188)	98.5 (79-257)	W = 252.5	0.306	
HbA1c (%), median (range)	5.7 (5-8.2)	5.85 (5.10-8)	W = 190.5	0.130	
Total cholesterol (mg/dL), mean ± SD	209.72 ± 37.94	206.27 ± 36.65	t = 0.821	0.417	
Triglyceride (mg/dL), mean ± SD	148.94 ± 59.33	132.33 ± 56.30	t = 1.967	0.057	
HDL (mg/dL), mean ± SD	51.91 ± 11.96	53.08 ± 14.26	t = −1.112	0.274	
LDL (mg/dL), mean ± SD	133.47 ± 32.22	133.61 ± 25.33	t = −0.032	0.975	
VLDL (mg/dL), mean ± SD	28.46 ± 11.75	26.91 ± 11.54	t = 1.024	0.314	
TAS (mmol Trolox equivalent/L), mean ± SD	1.55 ± 0.17	1.58 ± 0.15	t = −1.224	0.231	
TOS (µmol H2O2 equivalent/L), median (range)	30.76 (28.30-37.45)	29.91 (27.87-39.14)	W = 112.5	0.060	
OSI (AU), mean ± SD	20.18 ± 2.13	19.24 ± 2.37	t = 2.388	0.020	
7-KC (ng/mL), median (range)	38.95 (26.74-69.73)	41.25 (28.30-94.73)	W = 216.0	0.085	
C-triol (ng/mL)	31.45 (14.77-77.86)	28.26 (14.84-72.57)	W = 49.0	0.388	
8-iso-PGF2α (pg/mL), median (range)	116.76 (38.90-1135.10)	121.90 (57.14-897.44)	W = 301.0	0.610	
NfL (pg/mL), median (range)	66 (38.15-258.11)	62.66 (36.98-274.16)	W = 261.0	0.250	

Baseline serum calcium and PTH levels were evaluated for correlations with oxidative stress markers and NfL. No significant correlations were found between serum calcium or PTH levels and any of these parameters. Serum NfL levels were assessed before and after PTX to determine any correlation with oxidative stress markers. Before surgery, a negative correlation was observed between NfL and TAS (r = −0.370, p = 0.044). However, after surgery, NfL levels showed no significant correlation with any of these parameters (Table 3).

**Table 3:** Correlation of the parameters R = Spearman correlation coefficient; df = 34 (n = 36, df = n − 2) for all correlations. PTH: parathyroid hormone, TAS: total antioxidant status, TOS: total oxidative status, OSI: oxidative stress index, 7-KC: 7-ketocholesterol, C-triol: cholestan-3β,5α,6β-triol, 8-iso-PGF2α: 8-iso-prostaglandin F2α, NfL: neurofilament light chain

Parameters	R value	P value
Serum Calcium (Pre-surgery)		
TAS	− 0.301	0.107
TOS	− 0.293	0.116
OSI	0.136	0.473
7-KC	− 0.120	0.514
C-triol	− 0.175	0.307
8-iso-PGF2α	0.051	0.767
NfL	− 0.021	0.904
Serum PTH (Pre-surgery)		
TAS	− 0.134	0.479
TOS	− 0.305	0.102
OSI	− 0.067	0.724
7-KC	0.255	0.159
C-triol	− 0.386	0.414
8-iso-PGF2α	− 0.031	0.857
NfL	− 0.010	0.955
Serum NfL (Pre-surgery)		
TAS	− 0.370	0.044
TOS	0.031	0.870
OSI	0.305	0.101
7-KC	− 0.021	0.910
C-triol	− 0.079	0.649
8-iso-PGF2α	− 0.067	0.698
Serum NfL (Post-surgery)		
TAS	− 0.209	0.268
TOS	− 0.127	0.503
OSI	0.073	0.702
7-KC	0.031	0.867
C-triol	0.135	0.431
8-iso-PGF2α	0.065	0.705

## Discussion

This study is the first, as per our literature review, to simultaneously assess oxidative stress and neuronal damage in patients with PHPT. A reduction in overall oxidative balance was observed after PTX, as evidenced by a significant decrease in OSI. Although there was an increase in TAS and a decrease in TOS, these changes were not statistically significant, indicating that the observed shift in oxidative balance was not driven by uniform changes across individual oxidative stress markers.

Another study conducted by Abdulrahman et al. reported no changes in TAS levels but noted decreases in other oxidative stress markers [15]. Conversely, Deska et al. observed an increase in TAS, a reduction in TOS, and a decrease in OSI following PTX [14]. The discrepancies between these studies may be attributable to differences in sample sizes and in the magnitude of changes in PTH levels.

In the present study, neither oxysterol levels nor 8-iso-PGF2α levels changed significantly following PTX. Oxysterols are formed through oxidative modification of cholesterol and reflect lipid peroxidation, while 8-iso-PGF2α is a well-established marker of arachidonic acid oxidation. The lack of significant change in these markers may suggest that lipid-specific oxidative pathways are less responsive to the biochemical remission achieved by PTX within the six-month follow-up period, or alternatively, that the sample size was insufficient to detect modest changes.

Numerous studies have assessed the relationship between PHPT and neurocognitive functions. It has been shown that mild PHPT is associated with cognitive features affecting verbal memory and nonverbal abstraction that improve after PTX [2]. Similarly, another study reported improvement in long-term auditory memory, short- and long-term visual memory, visual attention, complex concentration skills, and executive abilities following PTX [21]. A study of 35 patients with PHPT reported improvements in visual memory, visual-constructive abilities, and direct memory one year after surgery [22]. In our study, NfL levels did not change after surgery. This finding may be partly explained by the slow biological turnover of neurofilaments, which suggests that six months may be insufficient for NfL levels to fully reflect neuronal recovery following biochemical remission. Additionally, neuronal injury in mild and/or asymptomatic PHPT may be subtle and chronic, potentially resulting in only modest baseline NfL elevations that are difficult to detect as statistically significant changes, particularly in a limited sample size. Longer follow-up periods and larger cohorts may be needed to capture meaningful longitudinal changes in NfL following PTX.

On the other hand, before PTX, a negative correlation was observed between TAS and NfL levels, which disappeared after surgery. This indicates that in PHPT, lower TAS levels are associated with higher NfL levels. Neurons, which have a higher density of mitochondria due to their substantial energy needs, are particularly susceptible to oxidative stress because mitochondria are a primary source of ROS [23]. The hippocampus, a brain region crucial for learning and memory, is especially vulnerable to oxidative damage [24]. Given that PHPT is associated with verbal memory impairments that tend to improve following surgical intervention, as supported by the studies above, it can be postulated that oxidative stress plays a contributory role in the neuronal damage observed in PHPT. As oxidative stress decreases following PTX, neuronal damage may also diminish. Another factor for increased neuronal injury may be increased intracellular calcium, which triggers calcium-dependent cascades that lead to neuronal cell death [25]. A reduction in intracellular calcium following surgery may lead to improvement in neuronal functions.

The reasons behind increased oxidative stress in PHPT remain largely unclear. Potential mechanisms include calcium-induced ROS production through increased mitochondrial metabolic rates and enhanced nitric oxide production [26]. Additionally, PTH may contribute to oxidative stress by elevating cardiac cell metabolism through its chronotropic and inotropic effects [27], and by stimulating mitochondrial β-oxidation of fatty acids in osteoblasts [28].

The study has some inherent limitations that warrant consideration; the foremost is the relatively small sample size, reflecting the number of consecutive eligible patients who presented to our clinic during the study period. Recruitment was further challenged by the COVID-19 pandemic and the requirement for two separate blood sampling visits, which limited patient participation. A formal a priori power calculation was not performed, as this is the first study to simultaneously assess oxysterol profiles, 8-iso-PGF2α, and NfL levels in PHPT, precluding reliable effect size estimation. The small sample size may have reduced the statistical power to detect significant differences in some parameters. For instance, the p value for the preoperative and postoperative difference in TOS was 0.06, indicating borderline statistical significance. A larger sample size might increase the likelihood of observing a statistically significant difference in TOS. Additionally, as noted above, the lack of change in individual oxidative stress markers may reflect that the overall shift in oxidative balance was not driven by uniform changes across individual biomarker pathways. Secondly, the absence of a control group limits the ability to compare oxidative stress and neuronal damage markers in PHPT patients to those in a healthy population or other disease groups. Furthermore, the present study should be regarded as exploratory and hypothesis-generating, and the findings require confirmation in larger, controlled trials. Lastly, although the pre-post design partially controls for inter-individual confounding factors such as age, BMI, smoking status, and comorbidities, residual confounding from variables that may change over the follow-up period, such as lifestyle changes, cannot be entirely excluded.

## Conclusions

PHPT is associated with elevated oxidative stress, which may contribute to neuronal damage. PTX appears to significantly reduce the overall oxidative balance, as reflected by a significant decrease in OSI; whether this reduction translates into better central nervous system outcomes remains a hypothesis that warrants confirmation in future studies with larger cohorts, longer follow-up periods, and neurocognitive assessments. Although oxidative stress is elevated in PHPT, the precise mechanisms responsible for this phenomenon are not entirely clear. Further preclinical and clinical research is needed to better comprehend the pathophysiology of PHPT and its effects on clinical outcomes.
